# Objectively Measured Physical Activity Levels and Associated Factors in Older US Women During the COVID-19 Pandemic: Cross-sectional Study

**DOI:** 10.2196/38172

**Published:** 2022-08-22

**Authors:** Renoa Choudhury, Joon-Hyuk Park, Ladda Thiamwong, Rui Xie, Jeffrey R Stout

**Affiliations:** 1 Department of Mechanical and Aerospace Engineering University of Central Florida Orlando, FL United States; 2 Disability, Aging and Technology Cluster University of Central Florida Orlando, FL United States; 3 College of Nursing University of Central Florida Orlando, FL United States; 4 Department of Statistics and Data Science University of Central Florida Orlando, FL United States; 5 School of Kinesiology and Physical Therapy College of Health Professions and Sciences University of Central Florida Orlando, FL United States

**Keywords:** physical activity, older women, COVID-19, sedentary behavior, wrist-worn accelerometers, ActiGraph, aging, elderly population, women's health, digital health, frail, healthy lifestyle

## Abstract

**Background:**

Physical activity (PA) is vital for attenuating the aging-related physiological and functional declines in women aged 60 years or above. However, little is known about the objectively assessed PA behavior in older women during the COVID-19 pandemic and its association with sociodemographics, health and physical function, and COVID-19 related factors.

**Objective:**

This study aims to examine the objectively measured PA levels and associated factors among older US women who were living under the physical distancing guidelines during the second year of the pandemic.

**Methods:**

In this cross-sectional study, we collected free-living PA data from 94 community-dwelling older women aged between 60 and 96 years (mean age 75.1 years, SD 7.3) using wrist-worn ActiGraph GT9X accelerometers between February and August 2021. We examined whether their daily duration spent in sedentary behavior (SB), light-intensity physical activity (LPA), and moderate-to-vigorous-intensity physical activity (MVPA) varied by sociodemographic characteristics, health and physical function, and COVID-19 related factors.

**Results:**

On average, participants accumulated 12.4 (SD 1.9) hours/day in SB, 218.6 (SD 64.3) minutes/day in LPA, and 42.4 (SD 31.0) minutes/day in MVPA, exhibiting overall reduced PA levels than previously published pre–COVID-19 norms of older US women. Among participants aged ≥80 years, sedentary time was 7.5% (*P*=.003) higher and the time spent in LPA and MVPA was, respectively, 13.3% (*P*=.03) and 44.9% (*P*<.001) lower than those aged 60-79 years. More MVPA participation and a less sedentary lifestyle were observed in those who had a higher self-rated health score (MVPA: *P*=.001, SB: *P*=.04) and lower fear of falling (FOF; MVPA: *P*=.003, SB: *P*=.04). Poorer performance in the 30-second sit-to-stand (STS) test was independently associated with more SB (*P*=.01) and less LPA (*P*=.04) and MVPA (*P*=.001) time among participants. In addition, sedentary time was 5.0% higher (*P*=.03) in frail and prefrail participants than their healthy counterparts.

**Conclusions:**

During the pandemic, older women spent the majority of their waking time being sedentary, while LPA accounted for a larger portion of their daily PA. Therefore, replacing SB with LPA (rather than MVPA) might provide a more feasible PA target for older women, particularly those aged ≥80 years or who have reduced physical function. In addition, targeted interventions might be beneficial in promoting an active lifestyle for those who live alone, are prefrail or frail, and have a high FOF in older age.

**International Registered Report Identifier (IRRID):**

RR2-10.2196/27381

## Introduction

### Background

The worldwide outbreak of COVID-19, caused by a novel type of coronavirus (SARS-COV-2), was declared a global pandemic by the World Health Organization (WHO) in March 2020. As of March 22, 2022, there were 470,839,745 COVID-19 cases confirmed worldwide, resulting in approximately 5,944,342 deaths [1]. Age and immune-compromised states are directly linked to the severity and fatality of COVID-19, making older adults the most significantly afflicted, particularly those with pre-existing health conditions (eg, chronic respiratory diseases, diabetes, hypertension, cardiovascular diseases, chronic kidney diseases) [2]. Hence, the Centers for Disease Control and Prevention (CDC) has been recommending strong adherence to physical distancing guidelines (previously known as social distancing guidelines) to the older adult population [3]. Despite the easing of stay-at-home order restrictions and advancement in the rapid, safe production and distribution of authorized vaccines, the physical distancing recommendations were still effective in the United States during the second year of the pandemic (ie, 2021), accompanied by the ongoing vaccination process and the emergence of new variants of concern (eg, Delta, Omicron).

However, practicing physical distancing may cause many older adults to limit their social interactions and out-of-home activities in community settings, which, in turn, is likely to affect their habitual physical activity (PA) level. Concerns regarding reduced PA levels resulting from COVID-19 mitigation strategies are particularly relevant for women aged 60 years or above, as they are the least active segment of the US population when evaluated against the current PA guidelines (ie, ≥150 minutes/week of moderate-to-vigorous-intensity physical activity [MVPA]). Federal monitoring data show that less than 20% of older US women were engaged in insufficient MVPA, even prior to the pandemic [4].

Therefore, it is important to examine the factors that may influence PA behavior in older women during the pandemic so that age- and gender-appropriate interventions can be tailored. To date, many studies have reported the factors associated with PA participation in older adults across different regions of the world during different phases of the pandemic, but the majority of these studies relied on self-report questionnaires and surveys for PA assessment [3,5-10]. Despite large and statistically robust sample sizes, these prior studies should be interpreted with caution because they can often be subjected to measurement biases (eg, social desirability bias and recall bias) and may not accurately capture the lower end of the PA spectrum (ie, light-intensity physical activity [LPA]) [11]. Objective PA measures can overcome these limitations of self-report questionnaires and provide continuous evaluation of 1 or more dimensions of PA (eg, frequency, intensity, and duration) in free-living conditions [12]. So far, a small number of studies have investigated objectively measured PA levels among Japanese, Swedish, and Brazilian older adults during the pandemic [13-16]; however, no study has yet reported objectively measured PA levels among older US people in the context of the pandemic.

Identifying nonmodifiable factors (eg, age, race/ethnicity, educational attainment, living alone) [17] associated with PA participation can help us in recognizing and targeting subgroups of older women who have been at higher risk of negative health consequences resulting from physical distancing adherence and are in most need of tailored PA interventions. Conversely, understanding the role of potentially modifiable risk factors (eg, overweight status, prefrail symptoms, fear of falling [FOF], upper and lower extremity strength) for reduced PA level during the pandemic might aid in developing evidence-based programs to enhance PA behavior in older women. Studies have reported obesity [18,19], frailty incidence [20], and decline in physical function (eg, upper and lower body strength) [21,22] to be associated with lower PA levels in older adults in the prepandemic period. In addition, FOF has been previously linked with more sedentary time and less duration in all other PA domains (both light and moderate-to-vigorous intensity) in a large cohort of older British men [23]. During the pandemic, older adults have been more vulnerable to social isolation and disconnectedness compared to the prepandemic time, due to changes in their lifestyle under the physical distancing guidelines. Given that social isolation poses a higher risk of frailty progression [24,25], increased FOF [26], and reduced physical functioning [27], it is important to understand how these factors are associated with different PA intensities among older adults in the light of physical distancing recommendations. This, in turn, will allow us to learn from this COVID-19 pandemic regarding PA strategies for older adults during social distancing and pandemic-related regulations in order to better prepare us for any similar instances possible in the future.

#### Goal of This Study

This study aims to examine PA levels among a diverse sample of older US women who were living under the physical distancing guidelines during the second year of the COVID-19 pandemic using wrist-worn accelerometry-based analysis. More specifically, we investigated whether their daily time spent in sedentary behavior (SB), LPA, and MVPA differed by (1) sociodemographic status (ie, age, race/ethnicity, education level, and household composition), (2) health and physical function (ie, BMI, self-rated health, frailty, FOF, grip strength, and sit-to-stand [STS] performance), and (3) COVID-19–related factors (ie, history of being COVID-19 positive, fear of COVID-19, and perceived severity of COVID-19 in their community).

## Methods

### Study Design and Participants

In this cross-sectional study, an opportunistic sample of 94 community-dwelling older women aged 60-96 years was recruited from the region of Central Florida, USA, between February and August 2021. Participants were recruited via word of mouth and flyers distributed in their communities. The inclusion criteria were that participants must be aged ≥60 years, be able to walk (with or without assistive devices but not requiring assistance from another person), have no marked cognitive impairment, live in their own homes or apartments, and be fluent in English or Spanish. The exclusion criteria were (1) having a medical condition that may preclude engagement in PA (eg, shortness of breath, dizziness, tightness or pain in the chest, and unusual fatigue at rest or with light exertion) and (2) currently receiving treatment from a rehabilitation facility. This cross-sectional assessment required 1 visit to the study site during which participants completed the informed consent, a self-report questionnaire, the Fatigue, Resistance, Ambulation, Illnesses, and Loss of weight (FRAIL) scale, and Short Falls Efficacy Scale-International (short FES-I), followed by the assessment of grip strength and STS performance. At the end of the visit, each participant was fitted with a wrist-worn accelerometer and given instructions on how to wear it during the PA-monitoring period.

### Ethical Considerations

The study protocol was approved by the Institutional Review Board of the University of Central Florida (Protocol No. 2189; September 10, 2020). All procedures were approved by the University of Central Florida’s Institutional Review Board (#00003029). All experimental procedures were performed in accordance with the University of Central Florida COVID-19 Human Subject Research Standard Safety Plan.

### Objective Measurement of PA

ActiGraph GT9X Link (ActiGraph LLC., Pensacola, FL, USA) was used to measure PA levels among participants. It is a small (3.5 × 3.5 × 1 cm), lightweight (14 g) wrist-worn device and contains a triaxial accelerometer with a dynamic range of ±8 gravitational units (g). Participants were required to wear it on the nondominant wrist for 7 consecutive days in free-living conditions. They were instructed to remove it only during sleeping, showering or swimming, and medical imaging tests. The accelerometer was initialized to record data at a sampling rate of 30 Hz. After 7 days of PA data collection, the ActiGraph devices were collected from the participants. At least 6 valid days of data were required for a participant to be included in the analysis, and only days during which the accelerometer was worn for at least 14 hours were counted as valid days.

Raw acceleration data from the ActiGraph devices were downloaded and converted to “.csv” files using ActiLife 6 v6.13.4 (ActiGraph LLC.). Next, data processing was performed in R statistical software (R Foundation for Statistical Computing) using the GGIR package (version 2.4-0) [28]. Data processing steps in GGIR include (1) autocalibration of acceleration signals according to local gravity [29], (2) detection of nonwear time, and (3) calculation of the average magnitude of dynamic acceleration corrected for gravity (ie, Euclidean Norm Minus 1 gravitational unit [g; ENMO]) over 5-second epochs, with negative values rounded to 0 [30]. ENMO was expressed in milligravitational units (mg) and defined as [31]:

ENMO (mg)=r_i_–1000

where 
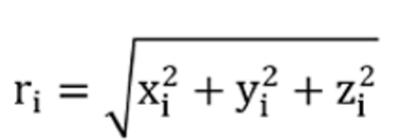
 is the i-th vector magnitude at each time point and 1 g=1000 mg.

Nonwear time and sustained abnormally high accelerations (ie, ≥5.5 g) were imputed using the default settings, described in detail elsewhere [30]. ENMO cut-off points were used to estimate the total time spent in SB, LPA, and MVPA among the participants. The following cut-off points for nondominant wrist-worn accelerometry for older adults were adopted from the literature [32,33]: (1) SB<30 mg, (2) 30 mg≤LPA<100 mg, and (3) MVPA≥100 mg. In addition, to understand the impact of the pandemic on PA levels among older women, our data were compared with 2 pre–COVID-19 observational studies that had large-scale older women population samples and used a similar protocol and data processing methods as ours [34,35].

### Assessment of Factors

A self-report questionnaire was used to obtain sociodemographic characteristics of participants. Based on age, they were categorized into 2 groups, *60-79 years* and *≥80 years*. According to race/ethnicity, participants were grouped into *White* and *non-White* groups, where the non-White category included African American, Asian, and Hispanic older women. The level of education was divided into 2 categories, *high school or lower* and *college or higher*. Household composition was defined as *living alone* or *living with family*. Height was measured using a stadiometer, and weight was measured using a digital scale. The BMI was calculated as weight (kg) divided by the square of height (m^2^). Based on BMI, the participants were categorized into *normal weight* (BMI<25 kg/m^2^), overweight (BMI=25-29.9 kg/m^2^), and obese (BMI≥30 kg/m^2^) [36]. Self-rated health was obtained using a 5-point Likert scale, and participants were classified into health status categories of *excellent* (score=1), *very good* (score=2), and *good or fair* (score≤3). Frailty was assessed using FRAIL scale, a 5-item self-report tool measuring fatigue, resistance, ambulation, illnesses, and loss of weight [37]. Based on the FRAIL score, participants were screened as *healthy* (score=0), *prefrail* (score=1-2), and *frail* (score=3-5). FOF was assessed using short FES-I, a 7-item self-administered tool measuring the level of concern about falling while performing 7 activities on a 4-point Likert scale (1=not at all concerned to 4=very concerned) [38]. A short FES-I score between 7 and 10 indicated a low concern of falling, while a score between 11 and 28 indicated a high concern of falling.

Grip strength, an indicator of hand and forearm muscle strength, was measured using a hydraulic hand dynamometer (JAMAR 5030J1, Patterson Medical), following the procedures adopted by the American Society of Hand Therapists, described in detail elsewhere [39]. Participants were categorized into *low* and *regular* grip strength groups based on the revised sarcopenia cut-off point (ie, <16 kg) recommended by the European Working Group on Sarcopenia in Older People (EWGSOP) [40]. The 30-second STS test (also known as the chair-stand test) was used to assess lower limb muscle strength, endurance, and balance among participants [39]. STS performance was divided into *below average*, *average*, and *above average* based on the age- and gender-specific normative scores provided by Rikli and Jones [41].

In addition, participants were asked whether they had ever tested positive for COVID-19. They also rated their perception of COVID-19 severity in their community over the past month on a 4-point Likert scale (1=extremely high, 2=moderately high, 3=severe, 4=not severe). Fear of COVID-19 among the participants was assessed using the Fear of COVID-19 Scale (FCV-19S), a 7-item, 4-point Likert scale adapted from Ahorsu et al [42]. An FCV-19S score between 7 and 21 was defined as normal fear of COVID, while a score between 22 and 35 indicated elevated fear of COVID.

### Statistical Analysis

All statistical analyses were performed using R statistical software (version 4.1.2) with a significance level (α) of .05. According to the Shapiro-Wilk test, SB and LPA among the participants were normally distributed, but MVPA showed nonnormal distribution. Descriptive statistics of PA variables (expressed as % of total wake time) were presented as means and SDs for normally distributed data and as medians and IQRs for nonnormally distributed data. For normally distributed PA variables, differences across 2 categories and more than 2 categories were examined, respectively, using independent *t* tests and 1-way ANOVA, with Bonferroni adjustment for post hoc comparisons. For nonnormally distributed variable, we performed the Mann-Whitney *U* test and the Kruskal-Wallis test, which are nonparametric equivalences of independent *t* tests and ANOVA, respectively.

Multiple linear regression analysis was performed on each PA outcome variable (ie, SB, LPA, and MVPA, expressed in minutes/day) to examine their adjusted associations with different independent variables. The independent variables included age, household composition, self-rated health score, frailty status, FOF score, and STS performance. For each PA outcome, model 1 was adjusted for the BMI and total wear time and model 2 was adjusted for the BMI and other 2 PA intensities. A priori sample size calculation revealed that the minimum required sample size for 9 explanatory variables at a statistical power level of 0.8 and a medium effect size (Cohen f^2^=0.2) would be 87; therefore, our sample size (ie, N=94) was sufficient for multiple regression. Before conducting the regression, multicollinearity was checked by examining the correlation matrix of independent variables for any correlation coefficient value >0.8. In addition, log_10_ transformation was performed on MVPA (minutes/day) in order to meet the linear regression assumption of normality of residuals. To aid interpretation, while presenting outcomes for MVPA models, regression coefficients were back-transformed using the formula 100×(exp^*β*^ – 1) to indicate the percentage change in MVPA (minutes/day) for 1-unit change in the corresponding independent variable [43].

## Results

### Participant Details

The mean age of participants was 75.1 (SD 7.3) years, and 23 (25%) participants were aged 80 years or above. The mean BMI was 26.85 (SD 5.42) kg/m^2^, and 39 (42%) participants were screened as prefrail. The mean grip strength was 19.0 (SD 5.6) kg, and the mean 30-second STS score was 14 (SD 6) repetitions (reps). The median accelerometer wear period for participants was 16.5 (IQR 15.5-17.6) hours/day. In addition, 85 (90%) participants had valid data (ie, ≥14 hours/day) on all 7 days. For the remaining participants (n=9, 10%), valid data were available for 6 days. All participants were included in the analysis. Among participants, the mean time spent in SB, LPA, and MVPA was 12.4 (SD 1.9) hours/day, 218.6 (SD 64.3) minutes/day, and 42.4 (SD 31.0) minutes/day, respectively. When expressed as a percentage of total waking time, the mean time accumulated in SB, LPA, and MVPA was 74.0% (SD 7.9%), 21.8% (SD 6.0%), and 4.2% (SD 3.0%), respectively.

**Figure 1:**
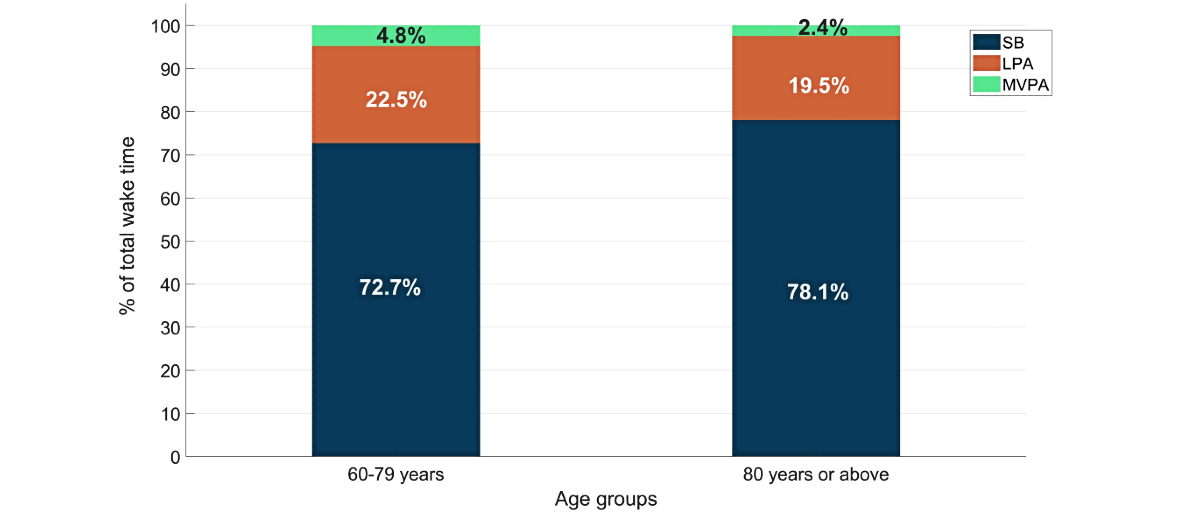
Distribution of mean PA levels by age group. LPA: light-intensity physical activity; MVPA: moderate-to-vigorous-intensity physical activity; PA: physical activity; SB: sedentary behavior.

Table 1 summarizes the descriptive statistics and the results from univariate analyses (parametric: independent *t* tests and 1-way ANOVA; nonparametric: Mann-Whitney *U* test and Kruskal-Wallis test) between PA variables and all factors. The average sedentary time was significantly higher (*P*=.003) in participants aged 80 years or above compared to those aged 60-79 years (78.10%, SD 7.49%, vs 72.70%, SD 7.54%), as shown in Figure 1. In addition, participants in the ≥80 years age group accumulated significantly less time in LPA (19.50%, *P*=.03) and MVPA (2.12%, *P*=.001) than those in the 60-79 years age group (LPA: 22.50%; MVPA: 3.85%). We observed that time spent in MVPA was significantly higher (*P*=.001) in participants who lived with their family compared to those living alone. However, no significant group differences were observed across race, education level, and BMI categories for any of PA variables in the current sample.

**Table 1:** Time spent in SB^a^, LPA^b^, and MVPA^c^ (expressed as % of total wake time), stratified by sociodemographic characteristics, health and physical function, and COVID-19–related factors.

Participant characteristics		Participants, n (%)	SB (%), mean (SD)	LPA (%), mean (SD)	MVPA (%), mean (SD)
Total		94 (100)	73.98 (7.85)	21.79 (6.04)	3.54 (3.53)
**Age (years)**					
	60-79	71 (75)	72.70 (7.54)	22.50 (5.84)	3.85 (3.50)
	≥80	23 (25)	78.10 (7.49)	19.50 (6.18)	2.12 (2.76)
	*P* value	N/A^d^	.003	.03	<.001
**Race**					
	Non-White	23 (25)	76.06 (7.73)	20.68 (6.03)	3.27 (1.61)
	White	71 (75)	73.31 (7.82)	22.14 (6.04)	3.90 (3.74)
	*P* value	N/A	.15	.32	.07
**Education**					
	College or higher	67 (71)	73.73 (7.39)	21.71 (5.44)	3.54 (3.69)
	High school or lower	27 (29)	74.62 (9.01)	21.97 (7.42)	3.36 (3.24)
	*P* value	N/A	.63	.85	.08
**Household composition**					
	Living alone	45 (48)	75.58 (8.58)	20.70 (6.5)	3.31 (3.07)
	Living with family	49 (52)	72.52 (6.87)	22.78 (5.46)	3.97 (3.43)
	*P* value	N/A	.06	.10	.001
**BMI^e^**					
	Normal weight	38 (40)	72.23 (7.12)	22.67 (5.21)	3.94 (2.80)
	Overweight	32 (34)	74.24 (8.84)	22.00 (7.11)	3.21 (3.83)
	Obese	24 (26)	76.41 (7.11)	20.10 (5.59)	2.96 (3.15)
	*P* value	N/A	.12	.26	.06
**Self-rated health**					
	Excellent	14 (15)	70.07 (7.98)	23.64 (5.46)	5.02 (5.13)
	Very good	36 (38)	73.18 (8.28)	22.04 (6.64)	3.98 (3.25)
	Good or fair	44 (47)	75.88 (6.99)	20.98 (5.67)	3.26 (2.29)
	*P* value	N/A	.04	.34	.001
**Frailty status**					
	Prefrail or frail	39 (42)	76.10 (7.70)	20.23 (5.79)	3.15 (3.57)
	Healthy	55 (58)	72.48 (7.66)	22.88 (6.02)	3.65 (3.22)
	*P* value	N/A	.03	.34	.06
**FOF^f^**					
	High	36 (38)	76.12 (8.76)	20.70 (7.20)	2.60 (3.80)
	Low	58 (62)	72.66 (6.97)	22.45 (5.14)	3.94 (3.45)
	*P* value	N/A	.04	.28	.003
**Grip strength**					
	Low (<16 kg)	40 (43)	73.21 (8.02)	22.77 (5.77)	3.46 (2.77)
	Regular (≥16 kg)	54 (57)	74.56 (7.74)	21.05 (6.18)	3.56 (3.55)
	*P* value	N/A	.42	.17	.39
**STS^g^ performance**					
	Below average	13 (14)	80.42 (7.22)	17.72 (6.12)	1.13 (1.57)
	Average	39 (41)	73.40 (8.15)	22.39 (6.60)	3.58 (3.46)
	Above average	42 (45)	72.56 (6.87)	22.48 (5.03)	3.99 (3.30)
	*P* value	N/A	.004	.03	<.001
**History of being COVID-19 positive**					
	No	87 (93)	74.01 (8.03)	21.66 (6.13)	3.54 (3.83)
	Yes	7 (7)	73.60 (5.37)	23.28 (4.84)	2.65 (1.15)
	*P* value	N/A	.89	.50	.42
**Fear of COVID-19**					
	Elevated fear	8 (9)	74.01 (5.48)	23.0 (4.73)	3.21 (1.26)
	Normal fear	86 (91)	73.98 (8.06)	21.67 (6.16)	3.57 (3.84)
	*P* value	N/A	.99	.56	.26
**Perceived severity of COVID-19 in community**					
	Severe or moderately severe	42 (45)	74.69 (7.54)	21.29 (6.01)	3.38 (2.54)
	Not severe	52 (55)	73.41 (8.11)	22.18 (6.09)	3.62 (4.13)
	*P* value	N/A	.48	.60	.51

^a^SB: sedentary behavior.

^b^LPA: light-intensity physical activity.

^c^MVPA: moderate-to-vigorous-intensity physical activity.

^d^N/A: not applicable.

^e^BMI: Body Mass Index.

^f^FOF: fear of falling.

^g^STS: sit-to-stand.

**Figure 2:**
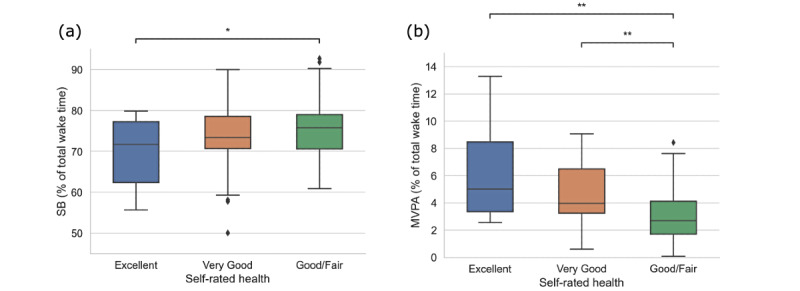
Time spent in (a) SB and (b) MVPA across categories of the self-rated health score. *P<.05 and **P<.01. MVPA: moderate-to-vigorous-intensity physical activity; SB: sedentary behavior.

The average sedentary time for participants with excellent health status was significantly lower than those rating their health as fair or poor (mean 70.07%, SD 7.98%, vs mean 75.88%, SD 6.99%, *P*=.01), as shown in Figure 2. Conversely, the mean time accumulated in MVPA was lower in participants with fair or poor health status (mean 3.26%, SD 2.29%) compared to those with excellent (mean 5.02%, SD 5.13%, *P*=.001) and very good (mean 3.98%, SD 3.25%, *P*=.04) health. We also found that sedentary time accumulated in prefrail and frail participants was significantly higher than that of participants with robust health (mean 76.10%, SD 7.70%, vs mean 72.48%, SD 7.66%, *P*=.03).

**Figure 3:**
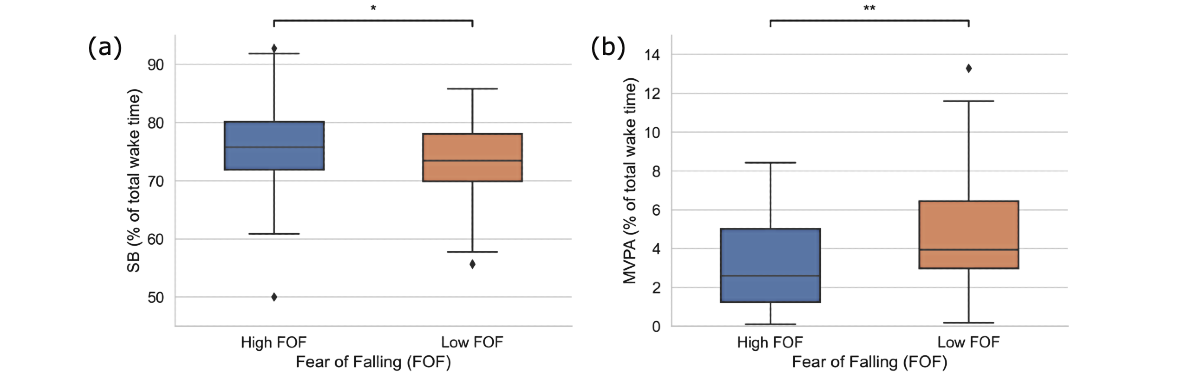
Time spent in (a) SB and (b) MVPA according to the FOF. *P<.05 and **P<.01. FOF: fear of falling; MVPA: moderate-to-vigorous-intensity physical activity; SB: sedentary behavior.

**Figure 4:**
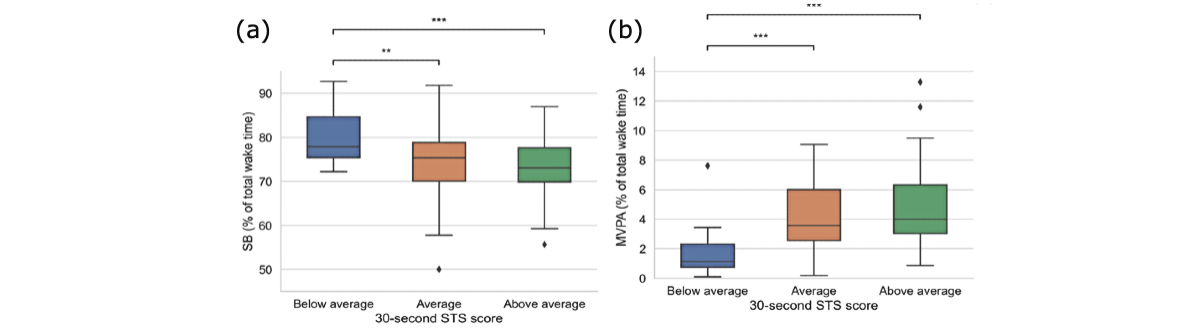
Time spent in (a) SB and (b) MVPA across categories of the 30-second STS score. **P<.01 and ***P<.001. MVPA: moderate-to-vigorous-intensity physical activity; SB: sedentary behavior; STS: sit-to-stand.

In the current sample, older women with low FOF participated more in MVPA (*P*=.003) and spent less time in being sedentary (*P*=.04) than their high-FOF counterparts (Figure 3). Regarding physical function, significant group differences were observed for STS performance but not for grip strength. Participants with below-average STS scores accumulated more sedentary time (mean 80.42%, SD 7.22%) and less MVPA time (mean 1.13%, SD 1.57%) than those with average (SB: mean 73.40%, SD 8.15%, *P*=.01; MVPA: mean 3.58%, SD 3.46%, *P*=.001) and above-average scores (SB: mean 72.56%, SD 6.87%, *P*=.001; MVPA: mean 3.99%, SD 3.30%, *P*<.001), as shown in Figure 4. In addition, time spent in LPA was significantly higher in participants with above-average STS scores compared to those with below-average scores (mean 22.48%, SD 5.03%, vs mean 17.72%, SD 6.12%, *P*=.01).

However, none of the COVID-19–related factors showed significant group differences for any of the PA variables. This might be attributed to the fact that only a small proportion of our study participants had a history of being COVID-19 positive and showed elevated fear of COVID-19 (ie, n=7, 7%, and n=8, 9%, respectively).

Table 2 presents the multiple regression models for sedentary time (minutes/day). In model 1, STS performance showed significant negative association (*β*=–3.92, *P*=.008) with the time spent in SB. This indicates that, in this current sample, an increase of 1-unit in STS score would result in a decrease of sedentary time by 3.92 minutes/day, after adjustment for total wear time and other variables. In model 2, after controlling for LPA and MVPA time, STS score did not show significant association with SB; however, FOF score was significantly positively associated with more time spent in SB (*β*=8.99, *P*=.01).

Results from multiple regression analysis for LPA (minutes/day) are reported in Table 3. In model 1 (adjusted for total wear time), STS performance had a significant positive association (*P*=.04) with LPA, indicating an increase of 2.39 minutes/day of LPA time for each 1-unit increase in the STS score. However, in model 2 (adjusted for SB and MVPA), no significant associations were observed between LPA and any independent variable.

Table 4 presents the back-transformed regression coefficients for MVPA models. In model 1, STS performance was significantly positively associated (*P*=.001) with MVPA, indicating a 5.13% change in MVPA time (minutes/day) for each 1-unit increase in the STS score. In model 2, after adjusting for SB and LPA time, the self-rated health score (*β*=20.92, *P*=.04) and STS performance (*β*=34.99, *P*=.02) showed a significant positive association with MVPA time.

Multimedia Appendix 1 reports the correlation matrix between all independent variables used in the linear regression analysis. All correlation coefficients were less than 0.8; therefore, no multicollinearity was detected.

**Table 2:** Association with SB^a^ (minutes/day): results from multiple regression analysis.

Participant characteristics		Model 1^b^			Model 2^c^	
		*β*^d^ (SE)	*P* value	*β* (SE)		*P* value
Age (years)		0.60 (1.14)	.60	–0.99 (1.84)		.59
**Household composition** (Ref.^e^: living alone)						
	Living with family	–12.27 (16.42)	.46	18.82 (26.36)		.48
Self-rated health score		–0.37 (10.67)	.97	8.42 (17.35)		.63
**Frailty status (Ref.: prefrail or frail)**						
	Robust health	–11.02 (16.78)	.51	–25.02 (26.62)		.35
FOF^f^ score		2.52 (2.32)	.28	8.99 (3.57)		.01
STS^g^ score (reps)		–3.92 (1.44)	.01	0.25 (2.42)		.92

^a^SB: sedentary behavior.

^b^Adjusted for the BMI and total wear time (minutes/day).

^c^Adjusted for the BMI, light-intensity physical activity (LPA; minutes/day), and moderate-to-vigorous-intensity physical activity (MVPA; minutes/day).

^d^*β*: standardized regression coefficient.

^e^Ref.: reference.

^f^FOF: fear of falling.

^g^STS: sit-to-stand.

**Table 3:** Association with LPA^a^ (minutes/day): results from multiple regression analysis.

Participant characteristics			Model 1^b^				Model 2^c^		
		*β*^d^ (SE)		*P* value		*β* (SE)		*P* value	
Age (years)			–0.13 (0.92)		.89		0.53 (0.83)		.53
**Household composition (Ref.^e^: living alone)**									
	Living with family	11.12 (13.20)		.40		2.74 (11.93)		.82	
Self-rated health score			–8.03 (8.58)		.35		–12.60 (7.72)		.11
**Frailty status (Ref.: prefrail or frail)**									
	Robust health	–12.72 (13.49)		.35		9.08 (12.04)		.45	
FOF^f^ score			–2.12 (1.87)		.26		1.28 (1.67)		.44
STS^g^ score (reps)			2.39 (1.16)		.04		0.50 (1.09)		.65

^a^LPA: light-intensity physical activity.

^b^Adjusted for the BMI and total wear time (minutes/day).

^c^Adjusted for the BMI, sedentary behavior (SB; minutes/day), and moderate-to-vigorous-intensity physical activity (MVPA; minutes/day).

^d^*β*: standardized regression coefficient.

^e^Ref.: reference.

^f^FOF: fear of falling.

^g^STS: sit-to-stand.

**Table 4:** Association with MVPA^a^ (minutes/day): results from multiple regression analysis.

Participant characteristics			Model 1^b^					Model 2^c^		
		*β*^d^ (SE)		*P* value		*β* (SE)			*P* value	
Age (years)			–1.98 (1.00)		.10		–1.78 (0.95)			.06
**Household composition (Ref.^e^: living alone)**										
	Living with family	31.0 (17.35)		.11		22.14 (15.03)			.14	
Self-rated health score			15.03 (11.63)		.20		20.92 (9.42)			.04
**Frailty status (Ref.: prefrail or frail)**										
	Robust health	–1.00 (18.53)		.94		–10.42 (15.02)			.43	
FOF^f^ score			–3.92 (2.02)		.11		–1.39 (1.92)			.46
STS^g^ score (reps)			5.13 (1.00)		.001		34.99 (1.21)			.02

^a^MVPA: moderate-to-vigorous-intensity physical activity.

^b^Adjusted for the BMI and total wear time (minutes/day).

^c^Adjusted for the BMI, sedentary behavior (SB; minutes/day), and light-intensity physical activity (LPA; minutes/day).

^d^*β*: standardized regression coefficient.

^e^Ref.: reference.

^f^FOF: fear of falling.

^g^STS: sit-to-stand.

## Discussion

### Principal Findings

This is the first study, to the best of our knowledge, to report the factors associated with objectively measured PA levels among a diverse sample of older US women during the COVID-19 pandemic. Our study findings indicate that participants spent the majority of their day being sedentary, confirming the high prevalence of a sedentary lifestyle among older adults reported in the literature [44]. Evidence suggests that a higher level of sedentary time remains associated with greater all-cause mortality risk among older adults, even among those who meet the national MVPA guidelines of 150 minutes/week [45]. We also observed that participants accumulated more time in LPA compared to MVPA, which confirms that LPA is the predominant form of PA behavior among women aged 60 years or above and accounts for a large portion of their daily activities [34]. Therefore, to combat a sedentary lifestyle, replacing SB with LPA (rather than MVPA) could be a more achievable target for older women, particularly those with chronic conditions and low cardiorespiratory fitness. Studies have reported LPA to be associated with reduced mortality risk, more favorable cardiometabolic biomarkers, and reduced incident mobility disorders in older adults [46-48]. Therefore, future studies should focus on identifying the optimal amount of LPA that may elicit health benefits in older women, irrespective of their engagement in MVPA, and developing LPA recommendations for the heterogeneous older adult population.

Compared to the previously published literature reporting normative PA levels of older US women in the prepandemic period using accelerometry-based analysis [49], our study participants’ PA levels were observed to be lower. Evenson et al [49] reported the average sedentary time in older US women, measured by hip-worn accelerometers, to be 510.6 (SD 98.8) minutes/day in the Women’s Health Study (WHS) cohort (n=16,726, mean age 71.5 years, SD 5.7 years, age range 62-89 years) and 555.6 (SD 99.4) minutes/day in the Women’s Health Initiative/Objective Physical Activity and Cardiovascular Health (WHI/OPACH) cohort (n=6126, mean age 78.7 years, SD 6.7 years, age range 63-97 years), which is less than the sedentary time among our participants (ie, mean 744, SD 114 minutes/day). Similarly, the average time accumulated in LPA (ie, mean 218.6, SD 64.3 minutes/day) and MVPA (ie, mean 42.4, SD 31.0 minutes/day) in our study sample was found to be less than the prepandemic PA norms reported in the WHS (LPA: mean 287.8, SD 54.63 minutes/day, MVPA: mean 91.9, SD 45.4 minutes/day) and WHI/OPACH cohorts (LPA: mean 286.9, SD 61.48 minutes/day, MVPA: mean 50.4, SD 34.4 minutes/day). These findings might qualitatively reflect the trend in PA and SB among older US women before and during the pandemic, despite the differences in accelerometer placement and PA intensity cut-off points. Previously, several studies have reported the negative impact of the pandemic on PA levels of older adults across different geographical regions during various phases of the pandemic [10]. Our results imply that despite the easing of stay-at-home orders in the United States, this trend of reduced PA participation among older women persisted during the second year of the pandemic, due to their adherence to the physical distancing guidelines. Previous findings suggest that physical distancing poses a risk of diminished social connectedness and can disproportionately impact older adults whose social interactions used to take place mostly outside the home (eg, community centers, volunteering services, places of worship), affecting their habitual PA level [50]. However, in this study, we were unable to quantify the longitudinal changes in PA behavior among our participants, since we did not have their free-living PA data from the prepandemic period.

This study also investigated the factors affecting the objectively measured PA and SB among older women during the pandemic. Our findings indicate that the time spent in SB increased with older age in our study sample. In general, older adults spend more time being sedentary than any other age groups [51], and our results support that even among older adults, older age is associated with a more sedentary lifestyle [52]. Prior studies have reported that age-related physiological and functional declines, as well as the prevalence of chronic diseases, may limit one’s ability to participate in MVPA in older age [53,54]. Therefore, it is important to identify the barriers to PA participation (eg, poor health, lack of knowledge, lack of motivation) in older women, particularly those aged 80 years or above, and provide individual-specific MVPA recommendations based on their aerobic capacity. For older women who cannot achieve 150 minutes/week of MVPA due to poor health and limited functional capacity, PA interventions incorporating both LPA and MVPA might provide a more feasible and sustainable approach in maintaining an active lifestyle [52].

In our study sample, lower MVPA time was observed in participants living alone compared to those living with family. During the pandemic, older women who live alone have been more susceptible to social isolation than in the prepandemic time, and the associations between social isolation and lower self-reported MVPA among older adults have been noted previously [55]. In addition, a recent study in Japan reported that during the third wave of the pandemic (ie, January 2021), recovery in the total PA time since the first wave (ie, April 2020) was observed among most Japanese older adults, except those who were living alone and were socially inactive [56]. This indicates that living with family might contribute to better resilience against the negative impact of the pandemic on the PA behavior of older women, because they are more likely to obtain valuable knowledge, support, and motivation from family members for maintaining a healthy lifestyle. In addition, this highlights the need for increasing social support for older women living alone in order to effectively promote MVPA participation among them.

Our findings suggest that better self-rated health was positively associated with less SB and more MVPA engagement in our study sample. Previously, existing studies have identified the bidirectional associations of SB and MVPA with self-rated health [57-59]. A study on middle-aged US adults reported that poor self-rated health is linked with adverse longitudinal shifts toward a more sedentary lifestyle and less MVPA time [58]. Conversely, Beyer et al [59] found that older individuals with positive self-perceptions of aging are more likely to participate in PA, which, in turn, improves their self-rated health. Therefore, PA intervention programs for older women should foster positive self-perceptions of aging, in conjunction with healthy lifestyle behaviors (eg, proper nutrition and diet intake, adequate sleep, no/reduced smoking and alcohol intake) for enhancing their PA participation so that they can achieve favorable self-rated health in later life.

Regarding frailty, our finding is consistent with the previous evidence that irrespective of MVPA participation, a higher level of SB is associated with the increased odds of being frail or prefrail in older age [60-62]. This also emphasizes the need for developing targeted interventions to reduce sedentary time among prefrail and frail older women, with a particular focus on decelerating or possibly preventing further functional loss in prefrail individuals.

FOF causes older adults to limit their habitual PA level, which, in turn, may increase their risk of falling more [23], and our result is in agreement with the existing literature [63,64]. Therefore, it is important to identify the barriers (both physical and psychological) to PA participation in older women with high FOF so that tailored interventions can be developed for those having irrational FOF despite having a low physiological risk of fall [65]. In addition, to reduce sedentary time in older women with high FOF and a high physiological risk of fall, focus should be given on integrated intervention approaches combining both cognitive behavioral therapy and balance exercises.

Our key findings indicate that STS performance was independently associated with all 3 PA variables (ie, SB, LPA, and MVPA time) in the regression analysis after adjusting for total wear time. The 30-second STS test is a widely used, well-validated functional performance measure in clinical research and practice, having good test-retest and interrater reliability [66]. STS performance is considered an indicator of lower limb strength among older adults and has been correlated with objective strength testing methods, such as leg-press resistance [67] and power rigs [68]. In our study, participants with below-average STS scores showed reduced PA levels compared to those with average and above-average scores. Previous findings have reported the bidirectional associations between SB and PA with lower body muscle strength [69], which is in agreement with our findings. In addition to lower body strength, STS scores have also been associated with dynamic balance and mobility [70] and are considered a proxy measure for physical performance in sarcopenia diagnosis [66]. These highlight the need for developing home-based multimodal intervention strategies during the pandemic to promote PA participation among older women, which will include (1) strength training for improving lower limb muscle mass and strength and (2) balance exercises for reducing the risk of falls.

Our focus was to identify the factors that were associated with higher sedentary time and less PA participation in our study sample during the pandemic. Based on our findings, considerations should be taken about an older individual’s age and health status and whether the person lives alone, is frail, and has high FOF and poor STS performance while providing PA prescriptions. For instance, if an older person has high FOF but good dynamic balance (ie, STS performance), the study result informs that the PA intervention should integrate approaches to reduce this irrational FOF for promoting PA participation. Again, if an older woman is more than 80 years old and has multiple chronic diseases, then PA intervention focusing on increasing LPA might be more effective and feasible (rather than increasing MVPA) to combat the sedentary lifestyle. These examples indicate how this knowledge of different factors associated with PA participation can contribute to individually tailored PA prescriptions for older women, rather than a one-size-fits-all approach, even during their transition to the postpandemic lifestyle.

### Strengths and Limitations

A strength of our study is the accelerometry-based measurement of the PA level during the pandemic, providing an objective and detailed description of SB, LPA, and MVPA patterns among women aged 60 years or above. In addition, identifying the factors associated with PA behavior provided evidence to develop informed strategies for maintaining or improving PA participation among older women in the context of the pandemic.

There are some recognized limitations of wrist-worn accelerometry-based studies, which apply to our study as well. For instance, wrist-worn accelerometers cannot accurately and reliably detect nonambulatory activities, such as resistance training or cycling. In addition, in some cases, a wrist-worn accelerometer can overestimate the PA level of the user while they perform activities that are primarily upper limb movements with low energy expenditure (eg, cleaning or sewing in a seated position). Furthermore, the cut-off points to classify PA intensity for wrist-worn accelerometers for older adults have not been firmly established yet. The nondominant wrist ENMO cut-off points for older adults, reported in the existing literature, range from 18 to 57 mg for the LPA threshold and 60 to 104 mg for the MVPA threshold [71-73], which limits the comparability of results among studies with different cut-off points. Moreover, due to the cross-sectional study design, we were not able quantify the change in PA behavior in our participants between the prepandemic period and the pandemic time, since we did not have their objectively measured pre–COVID-19 PA data. Another limitation of this study was the small, nonrepresentative nature of the sample. This sample was predominantly White, educated, relatively healthy, and active (75.6% meeting the national MVPA guidelines), which limits the generalizability of our findings.

### Conclusion

This study investigated objectively measured SB and PA in a sample of older US women during the COVID-19 pandemic. When compared to pre–COVID-19 norms of older US women, it was observed that the total time spent in LPA and MVPA was lower during the pandemic, while the average sedentary time was higher. A more sedentary lifestyle was found in participants who were aged 80 years or above, had poorer self-rated health, were frail or prefrail, and had high FOF. The time spent in LPA was significantly lower among women aged 80 years or above. Participation in MVPA was higher for those who were aged 60-79 years, lived with family, had better self-rated health, and had low FOF. In addition, it was observed that STS performance was independently associated with increased PA levels among participants after adjustment for total accelerometer wear time. These findings can help design more sustainable and behavior-changing PA interventions for older women to promote healthy aging and mitigate long-term health consequences of the pandemic.

## Appendix

Multimedia Appendix 1 Correlation matrix of variables used in the regression analysis.

## Abbreviations

CDC: Centers for Disease Control and Prevention
ENMO: Euclidean Norm Minus 1 g
FCV-19S: Fear of COVID-19 Scale
FES-I: Falls Efficacy Scale-International
FOF: fear of falling
FRAIL: Fatigue, Resistance, Ambulation, Illnesses, and Loss of weight
g: gravitational unit
LPA: light-intensity physical activity
mg: milligravitational unit
MVPA: moderate-to-vigorous-intensity physical activity
PA: physical activity
SB: sedentary behavior
STS: sit-to-stand
WHI/OPACH: Women’s Health Initiative/Objective Physical Activity and Cardiovascular Health
WHO: World Health Organization
WHS: Women’s Health Study

## References

[1] World Health Organization WHO Coronavirus (COVID-19) Dashboard.

[2] Shahid Z, Kalayanamitra R, McClafferty B, Kepko D, Ramgobin D, Patel R, Aggarwal CS, Vunnam R, Sahu N, Bhatt D, Jones K, Golamari R, Jain R (2020). COVID-19 and older adults: what we know. J Am Geriatr Soc.

[3] Callow DD, Arnold-Nedimala NA, Jordan LS, Pena GS, Won J, Woodard JL, Smith JC (2020). The mental health benefits of physical activity in older adults survive the COVID-19 pandemic. Am J Geriatr Psychiatry.

[4] Wegner L, Mendoza-Vasconez AS, Mackey S, McGuire V, To C, White B, King AC, Stefanick ML (2021). Physical activity, well-being, and priorities of older women during the COVID-19 pandemic: a survey of Women's Health Initiative Strong and Healthy (WHISH) intervention participants. Transl Behav Med.

[5] Carriedo A, Cecchini JA, Fernandez-Rio J, Méndez-Giménez Antonio (2020). COVID-19, psychological well-being and physical activity levels in older adults during the nationwide lockdown in Spain. Am J Geriatr Psychiatry.

[6] Suzuki Y, Maeda N, Hirado D, Shirakawa T, Urabe Y (2020). Physical activity changes and its risk factors among community-dwelling Japanese older adults during the COVID-19 epidemic: associations with subjective well-being and health-related quality of life. Int J Environ Res Public Health.

[7] Bailey L, Ward M, DiCosimo A, Baunta S, Cunningham C, Romero-Ortuno R, Kenny RA, Purcell R, Lannon R, McCarroll K, Nee R, Robinson D, Lavan A, Briggs R (2021). Physical and mental health of older people while cocooning during the COVID-19 pandemic. QJM.

[8] Wilke J, Mohr L, Tenforde AS, Edouard P, Fossati C, González-Gross M, Sánchez Ramírez C, Laiño F, Tan B, Pillay JD, Pigozzi F, Jimenez-Pavon D, Novak B, Jaunig J, Zhang M, van Poppel M, Heidt C, Willwacher S, Yuki G, Lieberman DE, Vogt L, Verhagen E, Hespanhol L, Hollander K (2021). A pandemic within the pandemic? Physical activity levels substantially decreased in countries affected by COVID-19. Int J Environ Res Public Health.

[9] Cheval B, Sivaramakrishnan H, Maltagliati S, Fessler L, Forestier C, Sarrazin P, Orsholits D, Chalabaev A, Sander D, Ntoumanis N, Boisgontier MP (2021). Relationships between changes in self-reported physical activity, sedentary behaviour and health during the coronavirus (COVID-19) pandemic in France and Switzerland. J Sports Sci.

[10] Oliveira MR, Sudati IP, Konzen VDM, de Campos AC, Wibelinger LM, Correa C, Miguel FM, Silva RN, Borghi-Silva A (2022). Covid-19 and the impact on the physical activity level of elderly people: a systematic review. Exp Gerontol.

[11] Marasso D, Lupo C, Collura S, Rainoldi A, Brustio PR (2021). Subjective versus objective measure of physical activity: a systematic review and meta-analysis of the convergent validity of the Physical Activity Questionnaire for Children (PAQ-C). Int J Environ Res Public Health.

[12] Strath SJ, Kaminsky LA, Ainsworth BE, Ekelund U, Freedson PS, Gary RA, Richardson CR, Smith DT, Swartz AM, Committee on Physical Activity of the American Heart Association Council on Lifestyle and Cardiometabolic Health (2013). Guide to the assessment of physical activity: clinical and research applications: a scientific statement from the American Heart Association. Circulation.

[13] Browne RA, Macêdo GAD, Cabral LL, Oliveira GT, Vivas A, Fontes EB, Elsangedy HM, Costa EC (2020). Initial impact of the COVID-19 pandemic on physical activity and sedentary behavior in hypertensive older adults: an accelerometer-based analysis. Exp Gerontol.

[14] Leavy B, Hagströmer M, Conradsson DM, Franzén E (2021). Physical activity and perceived health in people with Parkinson disease during the first wave of COVID-19 pandemic: a cross-sectional study from Sweden. J Neurol Phys Ther.

[15] Miyahara S, Tanikawa Y, Hirai H, Togashi S (2021). Impact of the state of emergency enacted due to the COVID-19 pandemic on the physical activity of the elderly in Japan. J Phys Ther Sci.

[16] Yamada Y, Yoshida T, Nakagata T, Nanri H, Miyachi M (2021). Letter to the editor: age, sex, and regional differences in the effect of COVID-19 pandemic on objective physical activity in Japan: a 2-year nationwide longitudinal study. J Nutr Health Aging.

[17] Prohaska T, Belansky E, Belza B, Buchner D, Marshall V, McTigue K, Satariano W, Wilcox S (2006). Physical activity, public health, and aging: critical issues and research priorities. J Gerontol B Psychol Sci Soc Sci.

[18] Zbrońska I, Mędrela-Kuder E (2018). The level of physical activity in elderly persons with overweight and obesity. Rocz Panstw Zakl Hig.

[19] Barreira T, Harrington D, Katzmarzyk P (2014). Cardiovascular health metrics and accelerometer-measured physical activity levels: National Health and Nutrition Examination Survey, 2003-2006. Mayo Clin Proc.

[20] Tolley AP, Ramsey KA, Rojer AG, Reijnierse EM, Maier AB (2021). Objectively measured physical activity is associated with frailty in community-dwelling older adults: a systematic review. J Clin Epidemiol.

[21] Mattioli RÁ, Cavalli AS, Ribeiro JAB, Silva MCD (2015). Association between handgrip strength and physical activity in hypertensive elderly individuals. Rev Bras Geriatr Gerontol.

[22] Rosenberg DE, Bellettiere J, Gardiner PA, Villarreal VN, Crist K, Kerr J (2016). Independent associations between sedentary behaviors and mental, cognitive, physical, and functional health among older adults in retirement communities. J Gerontol A Biol Sci Med Sci.

[23] Jefferis BJ, Iliffe S, Kendrick D, Kerse N, Trost S, Lennon LT, Ash S, Sartini C, Morris RW, Wannamethee SG, Whincup PH (2014). How are falls and fear of falling associated with objectively measured physical activity in a cohort of community-dwelling older men?. BMC Geriatr.

[24] Jarach C, Tettamanti M, Nobili A, D'avanzo B (2021). Social isolation and loneliness as related to progression and reversion of frailty in the Survey of Health Aging Retirement in Europe (SHARE). Age Ageing.

[25] Pizano-Escalante MG, Anaya-Esparza LM, Nuño Karla, Rodríguez-Romero JdJ, Gonzalez-Torres S, López-de la Mora DA, Villagrán Z (2021). Direct and indirect effects of COVID-19 in frail elderly: interventions and recommendations. J Pers Med.

[26] Nakamura M, Imaoka M, Nakao H, Hida M, Imai R, Tazaki F, Takeda M (2021). Increased anxiety about falls and walking ability among community-dwelling Japanese older adults during the COVID-19 pandemic. Psychogeriatrics.

[27] Del Pozo Cruz B, Perales F, Alfonso-Rosa RM, Del Pozo-Cruz J (2021). Impact of social isolation on physical functioning among older adults: a 9-year longitudinal study of a U.S.-representative sample. Am J Prev Med.

[28] Migueles J, Rowlands A, Huber F, Sabia S, van HV (2019). GGIR: a research community–driven open source R package for generating physical activity and sleep outcomes from multi-day raw accelerometer data. JMPB.

[29] van Hees VT, Fang Z, Langford J, Assah F, Mohammad A, da Silva ICM, Trenell MI, White T, Wareham NJ, Brage S (2014). Autocalibration of accelerometer data for free-living physical activity assessment using local gravity and temperature: an evaluation on four continents. J Appl Physiol (1985).

[30] van Hees VT, Gorzelniak L, Dean León EC, Eder M, Pias M, Taherian S, Ekelund U, Renström F, Franks PW, Horsch A, Brage S (2013). Separating movement and gravity components in an acceleration signal and implications for the assessment of human daily physical activity. PLoS One.

[31] Bakrania K, Yates T, Rowlands AV, Esliger DW, Bunnewell S, Sanders J, Davies M, Khunti K, Edwardson CL (2016). Intensity thresholds on raw acceleration data: Euclidean Norm Minus One (ENMO) and mean amplitude deviation (MAD) approaches. PLoS One.

[32] Suorsa K, Pulakka A, Leskinen T, Pentti J, Holtermann A, Heinonen O (2020). Comparison of sedentary time between thigh-worn and wrist-worn accelerometers. JMPB.

[33] Menai M, van Hees VT, Elbaz A, Kivimaki M, Singh-Manoux A, Sabia S (2017). Accelerometer assessed moderate-to-vigorous physical activity and successful ageing: results from the Whitehall II study. Sci Rep.

[34] LaCroix Andrea Z, Bellettiere John, Rillamas-Sun Eileen, Di Chongzhi, Evenson Kelly R, Lewis Cora E, Buchner David M, Stefanick Marcia L, Lee I-Min, Rosenberg Dori E, LaMonte Michael J, Women’s Health Initiative (WHI) (2019). Association of Light Physical Activity Measured by Accelerometry and Incidence of Coronary Heart Disease and Cardiovascular Disease in Older Women. JAMA Netw Open.

[35] Lee I-Min, Shiroma Eric J, Evenson Kelly R, Kamada Masamitsu, LaCroix Andrea Z, Buring Julie E (2018). Using Devices to Assess Physical Activity and Sedentary Behavior in a Large Cohort Study, the Women's Health Study. J Meas Phys Behav.

[36] Nuttall F (2015). Body mass index: obesity, BMI, and health: a critical review. Nutr Today.

[37] Morley JE, Malmstrom TK, Miller DK (2012). A simple frailty questionnaire (FRAIL) predicts outcomes in middle aged African Americans. J Nutr Health Aging.

[38] Kempen GIJM, Yardley L, van Haastregt JCM, Zijlstra GAR, Beyer N, Hauer K, Todd C (2008). The Short FES-I: a shortened version of the falls efficacy scale-international to assess fear of falling. Age Ageing.

[39] Thiamwong L, Stout JR, Park J, Yan X (2021). Technology-based fall risk assessments for older adults in low-income settings: protocol for a cross-sectional study. JMIR Res Protoc.

[40] Cruz-Jentoft AJ, Bahat G, Bauer J, Boirie Y, Bruyère O, Cederholm T, Cooper C, Landi F, Rolland Y, Sayer AA, Schneider SM, Sieber CC, Topinkova E, Vandewoude M, Visser M, Zamboni M, Writing Group for the European Working Group on Sarcopenia in Older People 2 (EWGSOP2)‚ the Extended Group for EWGSOP2 (2019). Sarcopenia: revised European consensus on definition and diagnosis. Age Ageing.

[41] Rikli R, Jones C (1999). Functional fitness normative scores for community-residing older adults, ages 60-94. J Aging Phys Act.

[42] Ahorsu DK, Lin C, Imani V, Saffari M, Griffiths MD, Pakpour AH (2020). The Fear of COVID-19 Scale: development and initial validation. Int J Ment Health Addict.

[43] Singer J, Willett J, Willett J (2003). Applied Longitudinal Data Analysis: Modeling Change and Event Occurrence.

[44] Gennuso KP, Gangnon RE, Matthews CE, Thraen-Borowski KM, Colbert LH (2013). Sedentary behavior, physical activity, and markers of health in older adults. Med Sci Sports Exerc.

[45] Chastin S, Gardiner P, Harvey JA, Leask CF, Jerez-Roig J, Rosenberg D, Ashe MC, Helbostad JL, Skelton DA (2021). Interventions for reducing sedentary behaviour in community-dwelling older adults. Cochrane Database Syst Rev.

[46] LaMonte MJ, Buchner DM, Rillamas-Sun E, Di C, Evenson KR, Bellettiere J, Lewis CE, Lee I, Tinker LF, Seguin R, Zaslovsky O, Eaton CB, Stefanick ML, LaCroix AZ (2018). Accelerometer-measured physical activity and mortality in women aged 63 to 99. J Am Geriatr Soc.

[47] Healy G, Dunstan D, Salmon J, Cerin E, Shaw J, Zimmet P, Owen N (2007). Objectively measured light-intensity physical activity is independently associated with 2-h plasma glucose. Diabetes Care.

[48] Glass NL, Bellettiere J, Jain P, LaMonte MJ, LaCroix AZ, Women’s Health Initiative (2021). Evaluation of light physical activity measured by accelerometry and mobility disability during a 6-year follow-up in older women. JAMA Netw Open.

[49] Evenson KR, Bellettiere J, Cuthbertson CC, Di C, Dushkes R, Howard AG, Parada H, Schumacher BT, Shiroma EJ, Wang G, Lee I, LaCroix AZ (2021). Cohort profile: the Women's Health Accelerometry Collaboration. BMJ Open.

[50] Armitage R, Nellums LB (2020). COVID-19 and the consequences of isolating the elderly. Lancet Public Health.

[51] Dogra S, Ashe MC, Biddle SJH, Brown WJ, Buman MP, Chastin S, Gardiner PA, Inoue S, Jefferis BJ, Oka K, Owen N, Sardinha LB, Skelton DA, Sugiyama T, Copeland JL (2017). Sedentary time in older men and women: an international consensus statement and research priorities. Br J Sports Med.

[52] Diaz K, Howard V, Hutto B, Colabianchi N, Vena J, Blair S (2016). Patterns of sedentary behavior in US middle-age and older adults: the REGARDS study. Med Sci Sports Exerc.

[53] Lee P, Cigolle C, Ha J, Min L, Murphy S, Blaum C, Herman WH (2013). Physical function limitations among middle-aged and older adults with prediabetes: one exercise prescription may not fit all. Diabetes Care.

[54] Volders E, de Groot RHM, Bolman CAW, Lechner L (2021). The longitudinal associations between change in physical activity and cognitive functioning in older adults with chronic illness (es). BMC Geriatr.

[55] Kobayashi LC, Steptoe A (2018). Social isolation, loneliness, and health behaviors at older ages: longitudinal cohort study. Ann Behav Med.

[56] Yamada M, Kimura Y, Ishiyama D, Otobe Y, Suzuki M, Koyama S, Kikuchi T, Kusumi H, Arai H (2021). The influence of the COVID-19 pandemic on physical activity and new incidence of frailty among initially non-frail older adults in Japan: a follow-up online survey. J Nutr Health Aging.

[57] Gregg E, Kriska A, Fox K, Cauley J (1996). Self-rated health and the spectrum of physical activity and physical function in older women. J Aging Phys Act.

[58] Gibbs BB, Sternfeld B, Whitaker KM, Brach JS, Hergenroeder AL, Jacobs DR, Reis JP, Sidney S, White D, Gabriel pk (2021). Bidirectional associations of accelerometer-derived physical activity and stationary behavior with self-reported mental and physical health during midlife. Int J Behav Nutr Phys Act.

[59] Beyer A, Wolff JK, Warner LM, Schüz B, Wurm S (2015). The role of physical activity in the relationship between self-perceptions of ageing and self-rated health in older adults. Psychol Health.

[60] Blodgett J, Theou O, Kirkland S, Andreou P, Rockwood K (2015). The association between sedentary behaviour, moderate-vigorous physical activity and frailty in NHANES cohorts. Maturitas.

[61] da Silva Coqueiro R, de Queiroz BM, Oliveira DS, das Merces MC, Oliveira Carneiro JA, Pereira R, Fernandes MH (2017). Cross-sectional relationships between sedentary behavior and frailty in older adults. J Sports Med Phys Fitness.

[62] Santos ISD, Silva CDFR, Ohara DG, Matos AP, Pinto ACPN, Pegorari MS (2021). Association between frailty syndrome and sedentary behavior among community-dwelling older adults in the Amazon region: a cross-sectional study. Sao Paulo Med J.

[63] Mendes da Costa E, Pepersack T, Godin I, Bantuelle M, Petit B, Levêque A (2012). Fear of falling and associated activity restriction in older people. Results of a cross-sectional study conducted in a Belgian town. Arch Public Health.

[64] Zijlstra GAR, van Haastregt JCM, van Eijk JTM, van Rossum E, Stalenhoef PA, Kempen GIJM (2007). Prevalence and correlates of fear of falling, and associated avoidance of activity in the general population of community-living older people. Age Ageing.

[65] Thiamwong L, Ng BP, Kwan RYC, Suwanno J (2021). Maladaptive fall risk appraisal and falling in community-dwelling adults aged 60 and older: implications for screening. Clin Gerontol.

[66] Yee XS, Ng YS, Allen JC, Latib A, Tay EL, Abu Bakar HM, Ho CYJ, Koh WCC, Kwek HHT, Tay L (2021). Performance on sit-to-stand tests in relation to measures of functional fitness and sarcopenia diagnosis in community-dwelling older adults. Eur Rev Aging Phys Act.

[67] Jones CJ, Rikli RE, Beam WC (1999). A 30-s chair-stand test as a measure of lower body strength in community-residing older adults. Res Q Exerc Sport.

[68] Hardy R, Cooper R, Shah I, Harridge S, Guralnik J, Kuh D (2013). Is chair rise performance a useful measure of leg power?. Aging Clin Exp Res.

[69] Buckinx F, Peyrusqué É, Granet J, Aubertin-Leheudre M (2021). Impact of current or past physical activity level on functional capacities and body composition among elderly people: a cross-sectional analysis from the YMCA study. Arch Public Health.

[70] Lord SR, Murray SM, Chapman K, Munro B, Tiedemann A (2002). Sit-to-stand performance depends on sensation, speed, balance, and psychological status in addition to strength in older people. J Gerontol A Biol Sci Med Sci.

[71] Sanders GJ, Boddy LM, Sparks SA, Curry WB, Roe B, Kaehne A, Fairclough SJ (2019). Evaluation of wrist and hip sedentary behaviour and moderate-to-vigorous physical activity raw acceleration cutpoints in older adults. J Sports Sci.

[72] Migueles JH, Cadenas-Sanchez C, Alcantara JMA, Leal-Martín J, Mañas A, Ara I, Glynn NW, Shiroma EJ (2021). Calibration and cross-validation of accelerometer cut-points to classify sedentary time and physical activity from hip and non-dominant and dominant wrists in older adults. Sensors (Basel).

[73] Fraysse F, Post D, Eston R, Kasai D, Rowlands AV, Parfitt G (2020). Physical activity intensity cut-points for wrist-worn GENEActiv in older adults. Front Sports Act Living.

