# Supplementary material for: Objectively Measured Physical Activity Levels and Associated Factors in Older US Women During the COVID-19 Pandemic: Cross-sectional Study
Source: JMIR Aging. 2022 Aug 22;5(3):e38172. doi: 10.2196/38172 (PMC9400844; doi:10.2196/38172)
Supplement: Multimedia Appendix 1 [file aging_v5i3e38172_app1.docx]

**Multimedia Appendix 1.** Correlation matrix of variables used in the regression analysis.

| Variables | Age | BMI | Household composition^a^ | Self-rated health^b^ | FRAIL score^b^ | Fear of falling score | Sit-to-stand score | Total wear time | SB | LPA | MVPA |
| --- | --- | --- | --- | --- | --- | --- | --- | --- | --- | --- | --- |
| Age | 1.00 |  |  |  |  |  |  |  |  |  |  |
| BMI | -0.05 | 1.00 |  |  |  |  |  |  |  |  |  |
| Household composition^a^ | -0.31** | -0.01 | 1.00 |  |  |  |  |  |  |  |  |
| Self-rated health^b^ | -0.12 | -0.34*** | 0.12 | 1.00 |  |  |  |  |  |  |  |
| FRAIL score^b^ | 0.13 | 0.26* | -0.17 | -0.36*** | 1.00 |  |  |  |  |  |  |
| Fear of falling score | 0.16 | 0.38*** | -0.25* | -0.50*** | 0.38*** | 1.00 |  |  |  |  |  |
| Sit-to-stand score | -0.32** | -0.36*** | 0.17 | 0.33** | -0.36*** | -0.40*** | 1.00 |  |  |  |  |
| Total wear time | -0.06 | 0.01 | 0.02 | -0.07 | 0.08 | 0.27** | -0.01 | 1.00 |  |  |  |
| SB | 0.08 | 0.16 | -0.10 | -0.21* | 0.20* | 0.18 | 0.40*** | 0.77*** | 1.00 |  |  |
| LPA | -0.15 | -0.20 | 0.17 | 0.07 | -0.18 | -0.17 | 0.32** | 0.27** | -0.34*** | 1.00 |  |
| MVPA | -0.26* | -0.20 | 0.15 | 0.41*** | -0.26* | -0.28** | 0.46** | 0.08 | -0.37*** | 0.43*** | 1.00 |

Note. **P* < .05, ***P* < .01 and ****P* < .001

Total wear time, SB, LPA and MVPA expressed in min/day

^a^Point-biserial correlation (living alone = 0, living with family = 1)

^b^Spearman's rank correlation. Rest of the table: Pearson correlation.
